# The Role of Pancreatic Enzyme Insufficiency in the Etiology of Functional Dyspepsia Resistant to Standard Treatment

**DOI:** 10.5152/tjg.2025.24729

**Published:** 2025-06-16

**Authors:** Fatih Kemik, Gozde Ceylan, Abdurrahman F. Aydin, Bilger Çavuş, Asli Ormeci, Ziya Imanov, Ibrahim V. Senkal, Kenan Nuriyev, Zulal Istemihan, Filiz Akyuz, Selman F. Besisik, Sabahattin Kaymakoglu, Kadir Demir

**Affiliations:** 1 Department of Internal Medicine, İstanbul University İstanbul Medical School, İstanbul, Türkiye; 2 Department of Biochemistry, İstanbul University İstanbul Medical School, İstanbul, Türkiye; 3 Department of Medicine, Division of Gastroenterology, İstanbul University İstanbul Medical School, İstanbul, Türkiye

**Keywords:** functional dyspepsia, functional dyspepsia resistant to standard treatment, pancreatic enzyme deficiency, pancreatic exocrine insufficiency

## Abstract

**Background/Aims:**

Functional dyspepsia (FD) is diagnosed in the absence of an identifiable organic cause. Pancreatic enzyme insufficiency (PEI) remains an underrecognized condition in these patients. This study aimed to investigate the prevalence of PEI among FD patients unresponsive to standard therapy and to evaluate its clinical and biochemical characteristics.

**Materials and Methods:**

A total of 154 patients diagnosed with FD were followed, among which 66 patients who did not respond to at least 4 weeks of standard treatment, including acid-reducing therapies, prokinetics, and antidepressants, were evaluated. Additionally, 34 healthy volunteers were included as a control group. Organic pathologies were excluded in all 66 patients with FD resistant to standard treatment using endoscopy, endoscopic biopsy, and imaging methods. Fecal elastase-1 (FE-1) enzyme levels were measured to determine the prevalence of PEI in both groups.

**Results:**

Pancreatic enzyme insufficiency was detected in 5 (7.57%) of the 66 treatment-resistant FD patients, while none of the controls had PEI. The prevalence of PEI was significantly higher in diabetic patients than in non-diabetic patients within the study group (*P* = .037). Patients with diarrhea, sticky stools, and frequent foul-smelling stools exhibited a higher prevalence of PEI (*P* = .022, *P* = .001, and *P* = .004, respectively). In the study group, PEI patients had lower serum calcium, phosphorus, and magnesium levels than the control group (*P* = .018, *P* = .011, and *P* = .001, respectively).

**Conclusion:**

Pancreatic enzyme insufficiency was identified in 7.57% of patients resistant to standard treatment. In patients resistant to standard therapy for at least 4 weeks, the presence of symptoms such as diarrhea, sticky stools, and foul-smelling stools, along with diabetes mellitus and low serum calcium, phosphorus, and magnesium levels, may warrant consideration of PEI as a potential underlying condition.

**Main Points**

- Exocrine pancreatic insufficiency (EPI) should be more frequently considered in the differential diagnosis of patients with *Helicobacter pylori*-negative functional dyspepsia resistant to standard treatment.
- Clinically, EPI should be suspected in patients presenting with diarrhea, sticky stools, foul-smelling gas/stool, and a concurrent diagnosis of diabetes.
- Decreased levels of serum vitamin D, calcium, phosphorus, and magnesium may accompany.

## Introduction

Dyspepsia is characterized by symptoms such as pain and burning in the epigastric region, bloating sensation after meals, early satiety, nausea, and vomiting. Its prevalence varies depending on the definition used in studies and the region investigated, with a prevalence of approximately 16%-20% in the general population.^1^ When investigating the etiology of dyspepsia, approximately 80% of patients do not have any organic pathology and are referred to as having functional dyspepsia (FD).^2^ Functional dyspepsia is a chronic condition that causes recurrent symptoms, leading to negative effects on patients’ quality of life and social functioning. The etiopathogenesis of FD is not fully understood, and there is no universally accepted treatment. Acid suppressants, prokinetic agents, and antidepressants have been used to treat FD, but their success rates are only about 40%-50%.^3^ Owing to its high prevalence, impact on the quality of life, and treatment cost, FD represents a significant economic burden.

Pancreatic enzyme insufficiency (PEI) is defined as a reduction in pancreatic enzyme activity in the intestinal lumen that is insufficient for normal digestion.^4^ The causes of PEI vary and can be classified as functional loss of the pancreatic parenchyma (chronic pancreatitis, cystic fibrosis, pancreatic resection, pancreatic tumors, fatty pancreas, etc.) and extra-pancreatic causes (celiac disease, diabetes mellitus (DM), gastric resection, Crohn’s disease, etc.).^5^^,6^ However, there are no reliable data on the prevalence of PEI.^7^ The clinical spectrum of PEI varies considerably among studies. Mild forms only exhibit dyspepsia symptoms such as abdominal pain and bloating, while severe PEI cases can result in steatorrhea, fat-soluble vitamin deficiencies, weight loss, and metabolic bone diseases, particularly when fat digestion and absorption are impaired.^8^ Nonspecific dyspepsia symptoms observed in mild cases generally do not suggest PEI in the differential diagnosis, which can lead to a delayed diagnosis. The fecal elastase-1 (FE-1) enzyme assay is an effective method and a widely accepted diagnostic test for detecting PEI.^9^^,10^ Although PEI is considered in the differential diagnosis of severe cases, mild cases are unfortunately overlooked, leading to a delayed diagnosis. However, after PEI diagnosis, pancreatic enzyme replacement therapy can prevent the symptoms and morbidity associated with PEI.

This study aims to investigate the clinical significance of exocrine pancreatic insufficiency (EPI) in patients with FD resistant to standard treatment. The main objective of this study is to assess the prevalence of EPI in patients with FD resistant to standard treatment. In addition to this primary objective, predictive clinical and biochemical parameters of EPI were explored in these patients to aid clinicians.

## Materials and Methods

### Study Group and Exclusion Criteria

A total of 154 patients who presented to the internal medicine and gastroenterology-hepatology clinics due to dyspeptic symptoms were evaluated in this study. The response to acid-suppressing agents, prokinetics, and antidepressants, administered for at least 4 weeks, was assessed. Sixty-six patients who continued to experience symptoms after treatment, with no organic pathologies detected through further diagnostic methods (endoscopy, endoscopic biopsy, and imaging) in addition to physical examination, were diagnosed with FD and included in the study. A total of 88 patients who responded to treatment or had organic pathologies detected through physical examination or imaging methods were excluded from the study. Additionally, 34 age and sex matched volunteers without clinical gastrointestinal symptoms were included. Written informed consent was obtained from all the patients and volunteers. The study was reviewed by the ethics committee of İstanbul Faculty of Medicine Clinical Research Ethics Committee and received ethical approval dated February 24, 2021, with the number 1048.

Patients and volunteers aged > 18 years were included in the study. Pregnant or lactating individuals, those with known pancreatic diseases (such as chronic pancreatitis, pancreatic cancer, or a history of pancreatic surgery), and those with known organic gastrointestinal pathologies were excluded from the study. Additionally, patients with peptic ulcer disease, gastric or duodenal malignancies, celiac disease, inflammatory bowel disease, eosinophilic gastroenteritis, and other structural or neoplastic gastrointestinal diseases identified via upper gastrointestinal endoscopy, endoscopic biopsy, or imaging methods (including abdominal ultrasound, computed tomography, or magnetic resonance imaging) were excluded. Furthermore, biliary tract diseases, cholelithiasis, chronic pancreatitis findings, liver cirrhosis, mesenteric ischemia, and pancreatic tumors detected through these imaging techniques were also considered exclusion criteria.

All included patients met the ROMA-4 FD criteria.^11^ The dyspepsia scores of the patients who were included in the study were determined and recorded using the Short-Form Leeds Dyspepsia Questionnaire (SF-LDQ).^12^ All patients planned for the study received standard dyspepsia treatment for at least 4 weeks, and only patients with FD who did not respond to the standard treatment were included in the study. None of the volunteers had gastrointestinal symptoms.

Stool samples were collected from the study participants to measure FE1, and the samples were stored at −80 °C in the laboratory until analysis. The collected fecal samples were prepared for analysis using a commercial stool preparation kit (Bioserv Diagnostics, Germany). Exocrine pancreatic function was evaluated in both groups using a commercial ELISA kit (Bioserv Diagnostics, Germany) that utilizes polyclonal antibodies to measure FE1 concentrations. Participants with FE1 > 200 μg/g were classified as having a normal exocrine function, those with ≥100 to <200 μg/g were classified as having mild to moderate exocrine PEI, and those with <100 μg/g were classified as having severe PEI.

### Treatment Regimens and Dosage Standardization

In this study, treatment regimens were determined based on established clinical guidelines for FD and PEI. The treatment was standardized by following evidence-based protocols for FD management, which include the use of proton pump inhibitors (PPIs), prokinetics, and antidepressants as first-line therapies when necessary. The dosages of these drugs were determined according to the manufacturer’s recommendations and the clinical needs of the patients, taking into account factors such as age, comorbidities, and the severity of symptoms. Specifically, PPIs were prescribed at a dose of 20-40 mg daily, prokinetics at 10 mg 3 times daily, and antidepressants at 10-50 mg (for Amitriptyline) as required. These regimens were followed for a minimum of 4 weeks before considering alternative diagnoses such as PEI. All treatment decisions were made by the attending physicians in accordance with the patient’s clinical condition and the best available evidence.

In this study, treatment resistance was defined as the persistence of dyspeptic symptoms in FD patients despite receiving at least 4 weeks of standard therapy, including PPIs, prokinetics, and, when necessary, antidepressants. This definition aligns with previous studies that have employed similar criteria for assessing treatment resistance in FD.^13^ Treatment resistance was determined based on patients’ symptom scores, as recorded using the SF-LDQ, which evaluates both the severity and frequency of dyspeptic symptoms. Patients who continued to experience significant symptoms despite appropriate pharmacologic intervention were classified as having treatment-resistant FD.

### Statistics

The obtained data were analyzed using appropriate statistical tests with SPSS 22.0 software (IBM SPSS Corp.; Armonk, NY, USA), and a *P*-value of <.05 was considered significant. Descriptive statistics are expressed as numbers, percentages, and 95% CI for categorical variables and as mean, SD, median, interquartile range, minimum, maximum, and percentile values for continuous variables. Subgroups were compared using chi-square (*Χ*^2^) or Fisher’s exact test for categorical variables, analysis of variance and/or student *t*-test for normally distributed continuous variables, and Kruskal–Wallis and/or Mann–Whitney *U* test for non-normally distributed variables.

## Results

### Demographic and Anthropometric Characteristics

In this study, 66 patients with FD and 34 sex- and age-matched volunteers who had no gastrointestinal symptoms were included (*P* = .76 and *P* = .79, respectively). Although the mean body mass index of patients was higher than that of volunteers, the difference was not significant (*P* = .17). Among these patients, 45.4% (n = 30) had comorbidities and 19.7% (n = 13) were diagnosed with DM. In contrast, 14.7% (n = 5) of the volunteers in the control group had comorbidities, and none were diagnosed with DM.

### Clinical Characteristics

**Figure 1.**
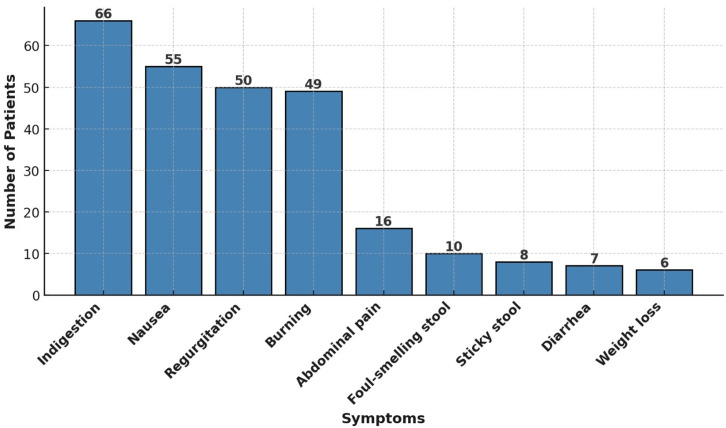
Prevalence of gastrointestinal symptoms in study participants.

The mean duration of dyspeptic symptoms in the patients was 27.4 ± 23.7 (range: 7-124) months. The presenting symptoms of the 66 patients with refractory dyspepsia who presented to the clinic are summarized in Figure 1.

### Pancreatic Enzyme Insufficiency Status

**Figure 2.**
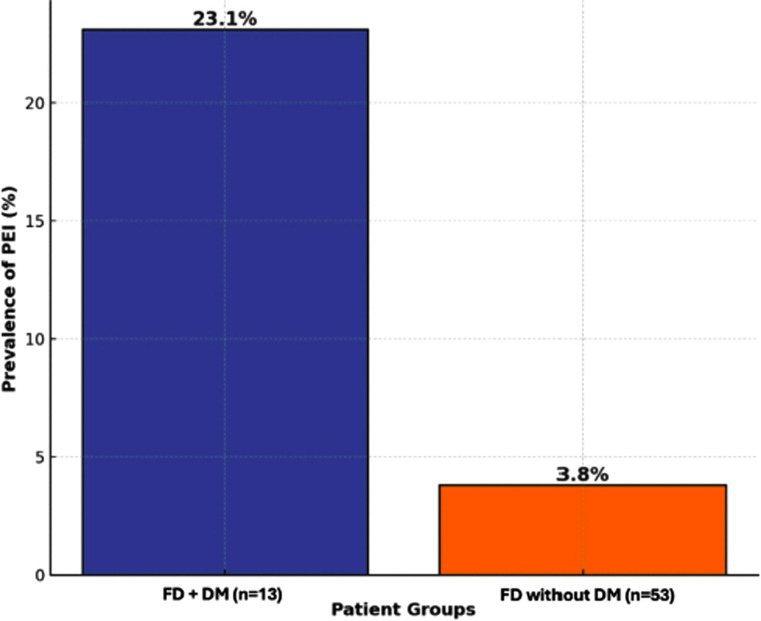
Prevalence of PEI in functional dyspepsia patients with and without diabetes mellitus. DM, diabetes mellitus; FD, functional dyspepsia; PEI, pancreatic exocrine insufficiency.

Among the 66 patients with FD resistant to standard treatment, 5 (one of them severe and 4 of them mild-moderate) were found to have PEI (7.57%). None of the 34 healthy volunteers developed PEI. Among the patients with FD and DM (13 patients), 3 had PEI (23.1%). Furthermore, only 2 of the 53 patients with FD and without DM had PEI (3.8%) (Figure 2). The frequency of DM in patients with FD with PEI resistant to standard treatment was significantly higher than that in patients with FD without PEI (*P* = .048). Diabetes mellitus was found to significantly increase the risk of developing PEI as an independent variable in patients with FD (*P* = .037). Of the patients with resistant FD, 15 (22.7%) were positive for *Helicobacter pylori* (*H*. *pylori*), while none of the 5 patients with PEI were *H*. *pylori*-positive.

In patients with FD with PEI, diarrhea was detected in 60% (n: 3), sticky stools in 80% (n: 4), and foul-smelling gas/stool in 80% (n: 4) of the patients, which was significantly higher than that in patients with FD without PEI (*P* = .022, *P* = .001, *P* = .004, respectively) (Table 1).

### Laboratory Characteristics

Compared to the control group, serum calcium, phosphorus, and magnesium levels were significantly lower in patients with FD with PEI (*P* = .018, *P* = .011, *P* = .001, respectively). Although vitamin D levels were not significantly different between the PEI and control groups, vitamin D levels were lower in the PEI group (mean values of 17.20 ng/mL and 26.67 ng/mL, respectively; *P* = .085).

## Discussion

Dyspepsia is a significant health problem that affects a considerable proportion of the global population. When evaluating hospital admissions for all causes, dyspepsia accounts for 2%-5% of all admissions.^14^ The etiology of dyspepsia is investigated using imaging methods, such as gastroscopy (colonoscopy if necessary), ultrasound, and computed tomography/magnetic resonance imaging; if no organic cause is found, a diagnosis of FD is made.^15^ Functional dyspepsia affects approximately 16%-20% of healthy individuals in the general population and is the most common functional gastrointestinal disease.^1^^,^^2^^,^^16^ Despite numerous studies on FD pathogenesis, particularly in the last 20 years, no definite cause has been found for FD.^17^

A recent study has shown that pancreatic diseases can play a role in the etiology of dyspepsia in up to 30%.^18^ In another study, PEI was detected in 5 (13.8%) of the 36 patients with FD, suggesting that this condition may lead to dyspeptic symptoms.^19^ In a recent double-blind randomized controlled trial involving 40 patients with FD, half of the patients received a multienzyme complex containing digestive enzymes, and the other half received a placebo for 2 months, during which dyspeptic symptom scores were recorded. Statistically significant decreases in dyspeptic symptom scores and clinical improvements were observed in patients receiving enzyme replacement therapy.^20^ Similarly, a recent consensus report emphasized the role of pancreatic diseases in patients presenting with dyspeptic symptoms, underlining the potential contribution of PEI in this context.^21^ Therefore, PEI should be considered in patients with FD. In this study, a subset of FD patients who were resistant to standard treatment was found to have PEI, suggesting a potential link between these conditions. In a different study conducted by Tahtacı et al,^22^ PEI was detected in 5 (14%) of 35 non-ulcer dyspepsia patients. The patients included in their study were those who were unresponsive only to oral acid-suppressive therapy and other potential organic pathologies were not excluded through imaging methods. The lower PEI rates in this study may be due to the characteristics of the selected patient group and the small sample size. Therefore, further studies with larger patient groups are required.

*Helicobacter pylori* is one of the most common bacterial infections in humans.^23^ Although the prevalence of *H. pylori* infection varies according to region and age, it is estimated to be 44.3% worldwide.^24^ Many studies have demonstrated the relationship between *H*. *pylori* and dyspepsia.^25^ *H*.* pylori* eradication therapy is commonly used to treat FD and has been shown to improve symptoms in some patients.^26^^,^^27^ In this study, *H*.* pylori* positivity was detected in 22.7% (n = 15) of the patients. The *H*.* pylori* positivity rate in the study group was lower than that in the average population. This might be because the majority of the patients in the study group continued to take PPIs during endoscopy, which can result in false-negative results for *H. pylori*, and that patients who had been symptomatic for a long time (mean symptom duration: 27 months) may have received *H. pylori* eradication therapy during this period. None of the patients with PEI in the study group were *H. pylori* positive. After excluding *H*.*pylori*-positive patients with FD who were resistant to standard treatment, the percentage of patients with PEI among *H*.*pylori*-negative FD patients was 10.41%, which is noteworthy. If a patient with standard treatment-resistant FD continues to have dyspeptic symptoms, they should be evaluated for PEI if *H. pylori* is negative.

Typical symptoms of dyspepsia include postprandial bloating, early satiety, epigastric pain, and epigastric burning.^28^ However, patients with PEI can present with various gastrointestinal symptoms. In this study, patients were questioned about the presence and severity of symptoms including diarrhea, sticky stools, foul-smelling stools, weight loss, abdominal pain, indigestion, regurgitation, burning, and nausea. It was observed that patients with PEI and FD resistant to standard treatment more frequently reported symptoms such as diarrhea, sticky stools, and foul-smelling gas/stools compared to those with FD without PEI, suggesting a possible association between PEI and stool abnormalities. Therefore, patients with dyspeptic symptoms should be enquired about diarrhea, sticky stools, and foul-smelling gas/stools. The presence of these complaints suggests that clinicians may consider PEI.

PEI is often accompanied by DM. As many factors such as the type and duration of diabetes, insulin requirement, presence of complications, and poor glycemic control can influence the development of PEI in diabetic patients, the frequency of PEI in diabetic patients varies despite being more common than in the general population.^29^^,^^30^ In this study, the prevalence of DM was higher in FD patients with PEI who were unresponsive to standard treatment compared to those without PEI. Furthermore, the presence of DM in FD patients who were resistant to standard treatment was found to be an independent risk factor for the development of PEI, highlighting the potential role of diabetes in the development of pancreatic exocrine insufficiency. When a patient with standard treatment-resistant FD has diabetes, PEI should be considered in the differential diagnosis.

In PEI, there may be deficiencies in fat-soluble vitamins, resulting in a decrease in serum vitamin D levels. In this study, the hematological and biochemical characteristics of patients were evaluated. Although no significant difference was observed in hematological parameters between the PEI and control groups. Serum calcium, phosphorus, and magnesium levels were notably lower in the PEI group. Vitamin D levels did not show a significant difference between the 2 groups, but the lower levels in the PEI group were particularly noteworthy, indicating a potential deficiency. The lack of significance may be because of the limited number of patients in this group. However, the low calcium and phosphorus levels in the PEI group compared to those in the control group may be explained by the low vitamin D levels. Recent extensive literature reviews suggest that low serum vitamin E levels, magnesium levels, and certain serum proteins (retinol-binding protein, prealbumin, etc.) may be helpful in the diagnosis of PEI and may guide the follow-up of pancreatic enzyme replacement therapy. In line with these findings, this study also found a significant decrease in serum magnesium levels in patients with PEI without gastrointestinal complaints compared with the control group.

Although this study is the largest cohort-based study conducted on this subject in the literature, the sample size is insufficient to reliably determine the prevalence of EPI in patients with FD. Further detailed studies with larger cohorts are needed on this topic. In this study, the prevalence of EPI was found to be higher in FD patients compared to healthy volunteers. However, another limitation of this study is the presence of 19.7% of patients with DM in the patient group, while no patients with a diagnosis of DM were included in the control group. The FD patients included in this study were meticulously selected and strict exclusion criteria were applied. In cases of small intestinal bacterial overgrowth and disaccharidase deficiencies, patients with clinically evident conditions were excluded. However, the absence of routine screening for these conditions can be considered a limitation of this study.

In patients with FD in whom organic pathologies have been ruled out by endoscopic and imaging methods, PEI may be considered an etiological factor if dyspeptic symptoms persist despite standard treatment. A detailed medical history should be obtained, and if there is accompanying diarrhea, foul-smelling gas/stool, or sticky defecation, PEI may be considered in the differential diagnosis. The presence of diabetes in these patients should increase the suspicion of PEI. Deficiencies in fat-soluble vitamins and serum levels of calcium, phosphorus, and magnesium should be considered as warning signs for PEI in patients with treatment-resistant FD. The accuracy of these findings should be further investigated in larger cohorts.

## Figures and Tables

**Table 1.** Clinical Characteristics of Patients with Functional Dyspepsia

Clinical Features	PEI+ (n = 5)	PEI- (n = 61)	*P*
Diarrhea	3 (60%)	7 (11.47%)	**.022**
Sticky stools	4 (80%)	6 (9.83%)	**.001**
Foul-smelling stool	4 (80%)	9 (14.75%)	**.004**
Weight loss	1 (20%)	5 (8.19%)	.389
Abdominal pain	3 (60%)	13 (21.31%)	.087

Bold values indicate statistically significant differences (*P* < .05). Values are expressed as n (%).PEI+, pancreatic enzyme insufficiency positive; PEI-, pancreatic enzyme insufficiency negative.

## Data Availability

All data and materials used in this study will be made available upon request through the corresponding author or via the designated data repository.
